# Injection-induced basement seismicity beneath the Raton Basin: constraints from refined fault architectures and basin structure

**DOI:** 10.1098/rsta.2023.0181

**Published:** 2024-08-09

**Authors:** Ruijia Wang, Evans A. Onyango, Brandon Schmandt, Lindsay Worthington

**Affiliations:** ^1^ Department of Earth and Space Sciences, Southern University of Science and Technology, Shenzhen, Guangdong, People's Republic of China; ^2^ Department of Earth & Planetary Sciences, University of New Mexico, Albuquerque, NM, USA; ^3^ Now at the Geophysical Institute, University of Alaska Fairbanks, Fairbanks, AK, USA

**Keywords:** waste water disposal, induced seismicity, Raton Basin, earthquake sources, receiver function

## Abstract

Intraplate earthquakes induced by anthropogenic fluid injection present unexpected seismic risk to previously quiescent or low seismicity-rate regions. Despite many studies of induced seismicity, there are relatively few with detailed openly accessible constraints on the interaction between seismic sources and subsurface structures. In this study of the Raton Basin, we refine source observations from a dense nodal array and constrain basin structure using teleseismic receiver functions. The cross-correlation-based relocated hypocentres and a new set of focal mechanisms light up active fault segments and show clear spatiotemporal patterns. The geometric complexity of reactivated fault clusters appears greatest near higher rate injection wells. Simpler normal fault structure is found farther from injection wells and near abrupt structural transitions suggested by receiver functions. While less induced seismicity in the crystalline basement is expected when injection is >1 km from the top of the basement (like Raton), our receiver function analysis identified a basin thickness ~3 km beneath the nodal array and lateral variations in sedimentary structures. Our results explain potential fluid connectivity between the injection depths focused at ~1–1.5 km below the surface and basement fault activity that begins at ~3 km and reaches peak activity at ~4–8 km depths.

This article is part of the theme issue ‘Induced seismicity in coupled subsurface systems’.

## 1. Introduction

Seismicity associated with anthropogenic activities is commonly observed around industrial operations that involve fluid injection or extraction, with earthquake magnitudes reaching 6 in rare cases [1], posing hazard to surrounding residences. Most of the M5+–induced seismicity occurred around long-term wastewater injection regions, like Oklahoma (Pawnee M5.8, Prague M5.7 and Fairview M5.1; e.g. [2–4]), Texas (Mentone M5; e.g. [5], potentially the M5.2 in November 2023, [6]) and Alberta (Peace River Mw5.1; e.g. [7–9]). Unlike injection with high pressures (e.g. hydraulic fracturing or geothermal exploration) that operate for short durations (e.g. weeks), wastewater injection is conducted at lower pressures but can be maintained for years to decades [10]. For the Raton Basin in the southwestern US, injection activities date back to the 1970s but injection increased substantially through the late 1990s and early 2000s [11], disposing of the wastewater associated with coal-bed methane extraction [12]. Spatially, injection wells span the majority of the basin with over 20 wells located within Colorado and ~7 in New Mexico, mostly targeting the Dakota Formation <2 km [13,14]. The monthly injection rate varies between the wells, ranging from ~10 000 barrels to over 30 000 barrels [15].

The seismicity distribution is heterogeneous across the basin: the largest event (Mw 5.3) occurred on the ~10-km-long Trinidad fault in 2011 [16], followed by years of quiescence along the fault zone [17]. Recent seismicity (2022–2024) in the basin is characterized by several M4+ on less-known faults towards the north. Station coverage remained sparse within the basin until the establishment of the YX array in 2016, which lowered the magnitude of completeness to ~M0.6 [15]. Although the potential for injection-induced seismicity is less debated than it was a decade ago, due to the well-documented rise of seismicity rates corresponding with injection at large-scale and over many years (e.g. [11,18]) the scarcity of accessible and co-located monitoring of seismicity and structural surveys still limit the understanding of the occurrence patterns of induced seismicity for many injection settings.

Previous monitoring and modelling of injection-induced seismicity have identified several interaction mechanisms between the external fluids and faults [1,19]. When fluid directly participates, pore pressure diffusion often initializes the activation of pre-existing faults [18,20], sometimes assisted by poroelastic effects (e.g. [21,22]). Other secondary mechanisms like thermal-elastic (e.g. [23,24]) and pressure surge (e.g. [25]) effects have been raised to explain various observations of seismicity related to injection, extraction and even remote triggering at volcanic settings (e.g. [26]). Despite the inconsistency between case studies, recent reviews emphasized that fluid still impacts activated faults primarily via pore-pressure diffusion or lowering the shear modulus [27,28]. Thus, under the assumption that faults have significant differential permeability compared to the host rock, the distribution of faults remains the primary factor controlling stress perturbation [29], and as such large-scale structures enable migration of pore fluid pressure.

Increasingly evidence for aseismic deformations has been identified around wastewater injection [30], hydraulic fracturing [31,32] and geothermal exploitation [33]. Such aseismic processes explain seismicity occurring beyond the spatial-temporal stress perturbation via pore-fluid pressure increase and poroelastic effects. In addition, it is generally believed that once activated, earthquake–earthquake interaction could dominate the occurrence of seismicity at later stages for both moderate-scale wastewater injection (e.g. [15]) and near-site hydraulic fracturing (e.g. [34]). Interestingly, the transition between the two endmember mechanisms is likely governed by fault roughness [35], mapping into the distribution of heterogeneity structures at a basin scale. From a depth view, decreasing the distance between the injection depth and top of the basement can increase the likelihood of induced seismicity occurrence [36], as suggested for Oklahoma [37], the Williston Basin, the Illinois Basin and the Appalachian Basin [38].

In summary, the static properties of fault zones and subsurface structures set the foundation for future dynamic reactions of faults under injection settings. So, an important aspect of understanding the basin-wide behaviour of induced seismicity lies within the basin structures between the injection depths and the faults within the deeper sedimentary layers or basement. To better understand the controlling structures as well as their contribution/reaction to fluid migration within the Raton Basin, we investigated both the earthquake source parameters and basin structures. The detailed geometries of recently active faults are unveiled by our cross-correlation-based relocated earthquake catalogue (i.e. hypoDD [39]) and 340 focal mechanisms from a dense short-term nodal-array monitoring. The source-based analyses are accompanied by new constraints on basin structure, revealed using teleseismic events recorded by both month-long nodal and 4-year broadband seismic arrays. With more detailed knowledge of activated faults and major transitions in basin structure, we can discuss potential drivers of the diverse seismic behaviour within the basin, aided by complementary information from the available injection and geologic well log records.

## 2. Geological setting and continuous seismic data

**Figure 1.**
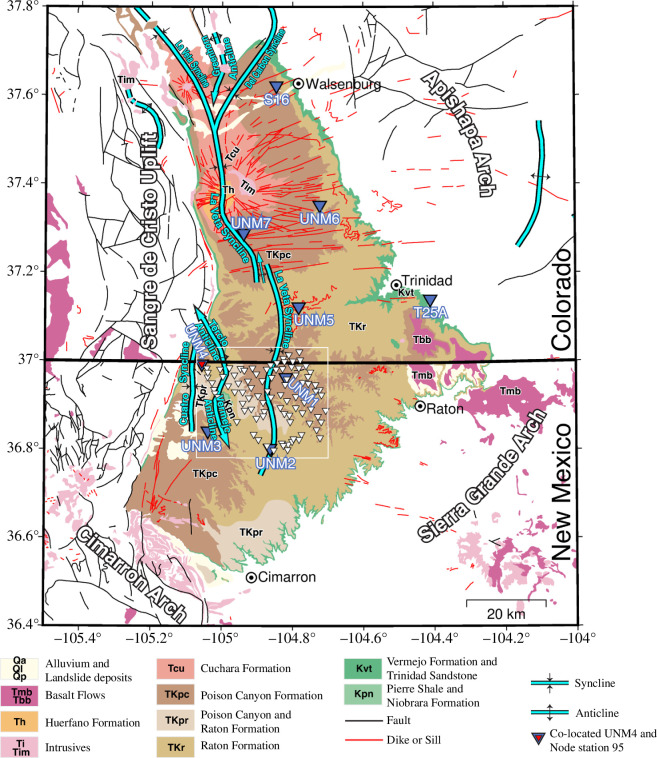
Geological structures of the Raton Basin (New Mexico geology from the Bureau of Geology and Mineral Resources, [40]); Colorado geology from Tweto [41] and the distribution of recent seismic monitoring arrays. Geologic units are labelled at the bottom. The broadband stations are denoted and labelled in dark blue and the nodal array stations are shown by the white triangles. The tiny red triangle near UNM4 marks a nearly co-located nodal station (95).

The Raton Basin is located at the Colorado–New Mexico border in the US, spans an area of less than 200 km^2^ and is roughly equally located in both states (figure 1). The basin is bounded by the Sangre de Cristo Uplift to the west, the Cimarron Arch to the south, Sierra Grande Arch to the southeast and the Apishapa Arch to the northeast (figure 1). A sequence of Devonian through Quaternary strata overlies the Precambrian basement [42]. Notable formations include the early Cretaceous Dakota formation, the late Cretaceous Trinidad sandstone and the late Cretaceous Raton formation. An array of igneous rocks and structures dominates the Colorado side of the basin: the largest igneous bodies are the two Spanish Peaks that stand >1900 m above surrounding relief [43]. An intrusive network of dikes radiates from the Spanish Peaks extending across the northern half of the basin and terminates at the eastern edge (i.e. red lines in figure 1). Extrusive basalt lava sheets cover the eastern tip of the basin near Trinidad, Colorado [44]. Estimated sediment thickness from a 10-km resolution map for North America [45] suggests a westward thickening of sediments up to ~4.5 km before abruptly decreasing at the western edge of the basin that is bounded by the Sangre de Cristo mountains. Wells directly sampling Precambrian basement are rare: based on well logs of Jurassic and younger strata and an estimate of the underlying Triassic and older sedimentary strata, Weingarten [46] estimated a shallower depth to basement for the Raton Basin with a maximum depth of ~3 km. New passive seismic data from within the basin during times of elevated seismicity could help clarify the depth to the basement and its proximity to seismogenic faults if hypocentre depths are tightly constrained.

**Figure 2.**
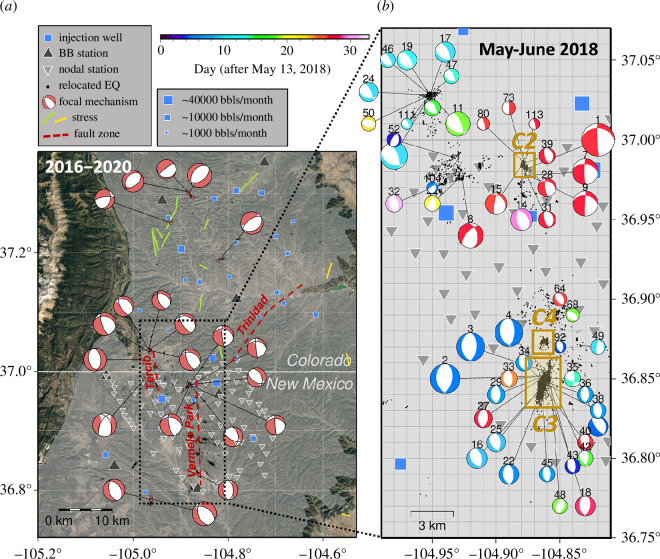
Regional fault zones, available earthquake catalogues and focal mechanisms. (*a*) 4-year catalogue from the broadband-coverage and focal mechanisms from waveform modelling (adopted after [15]). The rough locations of three fault zones are marked by the red dashed lines with names labelled. Monthly injection well data are publicly accessible and the well symbol sizes are in proportion to their injection volumes [13,14]. (*b*) 1-month catalogue and newly constrained focal mechanisms for selected 50 events (i.e. M > 1 and a few isolated/representative cases, indexed as in electronic supplementary material, figure S1) using the nodal array. The brown boxes mark the C2–C4 clusters that are better covered by the nodal array and investigated in detail.

Current stress status within the Raton Basin is dominated by a normal regime, evidenced by the 2013 M5.3 event within the Trinidad fault zone, recent focal mechanisms of M4+ events [15] and constraints from well measurements (i.e.[ 47]). The vertical stress is expected to be the largest across the basin, whereas the maximum horizontal stress turns from E–W at the east edge to N–S within the Colorado side (figure 2*a*).

The continuous waveforms used for this study come from two types of open-access seismic datasets (figure 2*a*). The first is a nodal array deployed in the central-to-southern section of the basin during the summer of 2018 that contains 96 three-component seismometers with 5 Hz geophones sampled at a rate of 250 Hz and an averaged inter-station distance of ~1–5 km (i.e. network code 4E). The second source of waveform data is longer-term (several years) broadband monitoring from nine broadband stations (i.e. network YX with seven stations and stations T25A, S16). All broadband stations provide a sampling rate of 100 Hz except S16, which recorded at 40 Hz. Broadband inter-station spacing ranges from ~15 to 30 km.

The nodal array was used for both source and receiver function (RF) analysis, whereas the broadband stations were primarily used in this study for teleseismic RF analysis over a longer operation period (>5 years). Our choice of using such hybrid data maximizes the utilization of available seismic data within the Raton Basin: the first-order subsurface structures remain stable over years, with seismicity evolves at a faster pace due to earthquake interactions and industrial operations over months. Finally, longer-term earthquake catalogues using the broadband array are the subject of other recent studies and are compiled in this study (e.g. figure 2*a*). For more in-depth analysis of multi-year evolution of seismicity recorded by the broadband array, one may refer to Glasgow *et al*. [15] and [17].

## 3. Method

### (a) Resolving the geometry of activated faults using refined location and focal mechanisms

The initial nodal array catalogue is adopted after Wang *et al*. [48], which took advantage of a machine-learning-based picker (i.e. PhaseNet [49]) and catalogue building workflow (i.e. now published and named as LOC-FLOW [50]). To detail the spatial–temporal evolution of the clusters and better constrain the fault structures, here we conducted two further analyses.

— We first calculated the differential times between the previous relocated events (i.e. [51]) via waveform cross-correlation (see FDTCC included in LOC-FLOW). A filter from 4 to 50 Hz was applied, and the time windows were defined as 0.1 s before and 0.5 s after the P or S phases. A minimum cross-correlation coefficient of 0.75 was required. We obtained a total of 2 075 464 pairs of differential times from 9401 events, which are further analysed using hypoDD [39] for relative location refinement (electronic supplementary material, figure S1).
— We manually picked the P-wave first motion polarity from the vertical component waveforms of the 96 stations (filtered between 1 and 40 Hz). We tried both FOCMEC [52] and HASH [53] for focal mechanism analysis and obtained comparable results. Considering the relatively good station coverage, P/S ratios are not used during the inversion with HASH. It is worth noting that adding S polarity or energy may not significantly improve the fitting since potentially large incident angles are expected within such a local scale array. Among the 378 picked events (*M* ≥ 0.5), 340 were resolved with a focal mechanism (see electronic supplementary material, figures S2 and S3), with 44% (i.e. 151) located along a major north-striking normal fault beneath the southern half of the node array, proportional to the number of detections (figure 2*b*).

### (b) Subsurface constraints from teleseismic receiver functions

Broadband receiver function analysis used 299 teleseismic events with *M* > 6 and epicentral distances of 25–99 degrees (electronic supplementary material, figure S4*a*). Minimum signal-to-noise ratios (SNR) of 10 and 5 were required for the vertical and north components, respectively. For the nodal array, due to the brief recording of only 34 days, we relaxed the signal strength requirements down to *M* > 5.0, SNR of 2 and 1.25 correspondingly, yielding six events suitable for further receiver function analysis on the nodal stations (electronic supplementary material, figure S4*b*).

**Figure 3.**
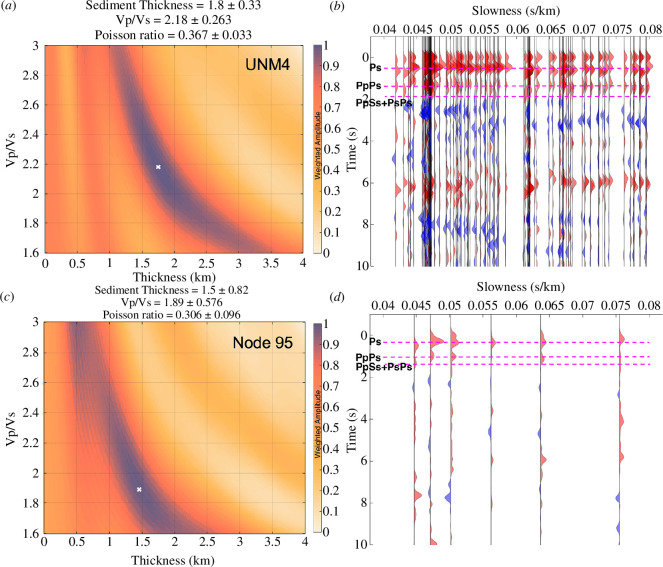
Examples of broadband and nodal receiver function analysis. (*a*) Plot of the H-κ search space 214 (grid increment = 0.1) for UNM4 with the optimal values marked by the white x. (*b*) Radial-component 215 receiver functions used in the stack for broadband station UNM4 are sorted by the slowness and shown 216 for a dominant frequency of 2.5 Hz. See Figure 1 for the location of UNM4. The best estimates of the 217 phase arrivals from H-κ stacking are labeled with dashed magenta lines. (*c*–*d*) same as (*a–b*) but for co-218 located nodal array station number 95.

Conventional teleseismic receiver function processing steps were implemented in the open-source RF software package of Eulenfeld [54]. Specifically, after removing the instrument responses, mean and trend, filtering (bandpass 1–20 Hz), as well as rotating to the vertical, radial and transverse coordinates, the vertical component at each station was deconvolved from the horizontal component using iterative time-domain deconvolution [55], producing an estimate of the Green’s function at the receiver (i.e. station) for a steeply incident source and providing sensitivity to major velocity contrasts at depth that would produce scattered phases in the teleseismic P coda. Finally, a moveout correction was applied based on a local one-dimensional velocity model [11] that has been adopted for hypocentre estimation (e.g. [15,48]). The individual receiver functions were stacked by epicentral distances with the time axis converted to depth (e.g. [56]). The final step in our analysis for each station involved a classic grid-search metric for optimal Vp/Vs ratio and structural layer thickness (i.e. H–κ [57]). In contrast to many studies focusing on the Moho, we focused on constraining the thickness of a low-velocity upper crustal layer (depths 0–4 km) that may provide insight into the depth to the basement (e.g. [58]). Given the shallow structural target, we stacked receiver functions calculated at relatively high frequencies (2.5 and 5 Hz) and searched over a wide range of potential Vp/Vs ratios between 1.6 and 3.0 (figure 3). An average sedimentary layer absolute Vp of 4.2 km/s was assumed based on the one-dimensional velocity model of Rubinstein *et al*. [11].

## 4. Results

### (a) New fault features within the updated catalogue

**Figure 4.**
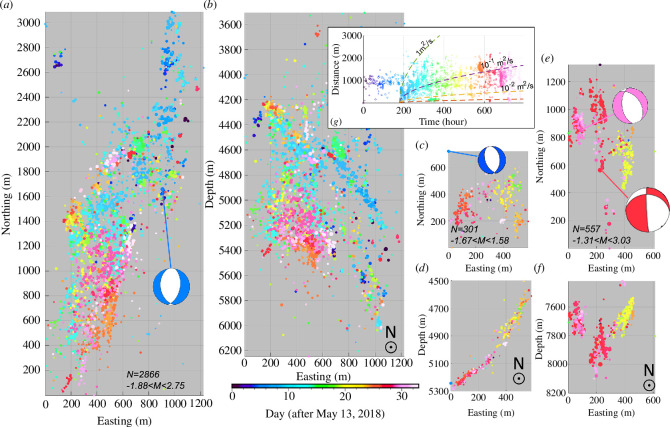
Zoom-in map and depth profiles showing the three clusters after waveform cross-correlation-based relocation: (a–b) C3, (c–d) C4, (e–f) C2. All figures are plotted at the same scale to enable comparison of relative fault sizes. The ‘beachballs’ indicate the focal mechanisms of representative earthquakes (i.e. event ID = 2, 92, 14 and 1 in figure 2*b*). Note that the depths relative to the surface instead of averaged sea level, are consistent with the receiver function analysis. (*g*) Diffusion rate modelling of C3 with the events colour-coded the same as in (*a*). Note the burst of clustered events at ~200 and ~700 h; the event time and location for the first event within the C3 cluster is used as the origin (0 h and distance).

The spatial distribution of earthquake hypocentres forms several clusters, in agreement with a previous study [48] but with higher resolution due to the use of cross-correlation-based differential times. Specifically, we are able to reveal the detailed three-dimensional fault geometries with less smearing along the depth (figure 4). These redefined earthquake hypocentres separate into roughly four clusters (C1–C4, also see [48]) defining fault geometries that are consistent with the focal mechanisms, offering an observational-based opportunity to visualize the basement fault segments active within the Tercio and Vermejo Park fault zones during the time of the node array deployment in 2018.

Here, we focus on the three tightly distributed clusters (C2, C3 and C4) that are better covered by the nodal array and exhibit clear linearity and spatial–temporal evolution (figure 4). All three clusters are located within the previously identified Vermejo Park fault zone [59]. As indicated by the relocated number of events and maximum magnitude, the southernmost C3 is the largest cluster that hosted six *M* > 2 events. The imaged fault extends from ~4 to ~6 km and dips ~45° towards the east (figure 4b). Events in C3 also spanned over the entire nodal array monitoring period (i.e. May–June 2018), with earlier events at shallower depth and then migrating downward to the east and south. As expected, the focal mechanisms are dominated by a normal faulting regime showing highly consistent strikes from the event distribution (figure 4a), focal mechanisms (electronic supplementary material, figure S2), and previous full moment tensors resolved with surface waveforms (figure 2 [15,48]). About 2 km north of C3, a 1-km-long fault was identified via the relocation (figure 4c). This newly identified west-dipping fault segment contains only 301 events that occurred within ~2 weeks. Despite a similar depth and location with C3, the migration pattern and activation time for C4 are significantly different from those of C3, with the earliest events only lighting up the shallower part (4.5–5 km, dip ~ 50°, figure 4d). Later events clustered along the down-dip direction at depths between 5 and 5.5 km (dip ~ 45°). Only one focal mechanism can be resolved next to this cluster, showing an NNW-striking normal fault.

Both the C3 and C4 clusters are located along the La Veta Syncline, relatively far from wastewater injection wells (~10 km). Further to the north and east, scattered events are present on both sides of the syncline with potentially normal faulting regimes as well (figure 2 and electronic supplementary material, figure S2). No events were observed further north until a deeper (7.5–8 km), dip-slip-dominated cluster (C2, figure 4e) showed up near the boundary between New Mexico and Colorado; normal events also exist within C2, likely from the subcluster located in the west. Although C3 and C4 clusters are resolved with similar normal faulting focal mechanisms, the spatial distribution suggests that their dipping angles are almost perpendicular to each other (figure 4).

Finally, the apparent diffusion rates are abnormally high (i.e. between 10^–1^ m^2^/s and 1 m^2^/s; Figure 4g) compared to those measured for sandstone samples in laboratories (e.g. 10^–8^ m^2^/s [60]). Such fast migration, if driven by fluid diffusion, is also beyond a reasonable geomechanical diffusion rate, and only possible for matured highly conductive fault zones (e.g. [29]). Considering the strong clustering (electronic supplementary material, figure S1) of all the seismicity, we suggest that pore pressure diffusion initiates seismicity, with subsequent spatiotemporal evolution dominated by earthquake–earthquake triggering.

### (b) Major reflectors from the RF analysis

After stacking at each station, receiver functions calculated from both the broadband and nodal arrays show the characteristic delayed sediment direct P arrival at low frequency (electronic supplementary material, figure S5), associated with sedimentary environments, which separates into direct P and following Ps phases for receiver functions calculated at higher frequencies [58,61]. Later phases observed correspond to a PpPs at ~1.2 s, a PpSs + PsPs at ~2 s, as well as the Ps signal from Moho reflection at ~6 s for broadband stations (figure 3b). Although the Moho conversion is not clearly obtained from the receiver functions of nodal seismometers with only <6 events, shallower signals in the nodal receiver functions are highly consistent with those from co-located broadband stations (figure 3c). For the H-k analysis of both broadband and nodal arrays, our constraint on the Vp/Vs is less certain than that for the thickness of the upper crust layer.

**Figure 5.**
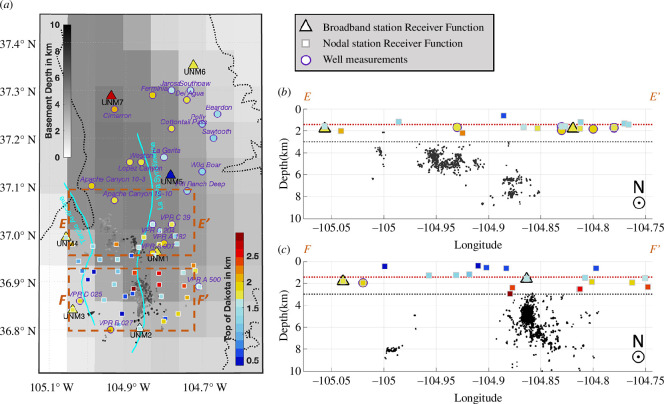
Map (*a*) and depth views of the resolved depths for the top of the Dakota Formation (except UNM5). In the map view, the dashed-black line indicates the boundary of Raton Basin and the basement depths from Mooney & Kaban [45] and shown in a grey scale (colourbar to the upper left). Events, wells and seismometers within the dotted boxes are projected to transect E–E′(*b*) and F–F′(*c*), respectively. Top of Dakota (red dotted lines) inferred from the mean of the wells and seismometer estimates that are projected to the line. Top of basement (grey dotted lines) inferred assuming a cumulative thickness of 1.438 km (e.g. [46]) between the top of Dakota and the top of the basement.

**Figure 6.**
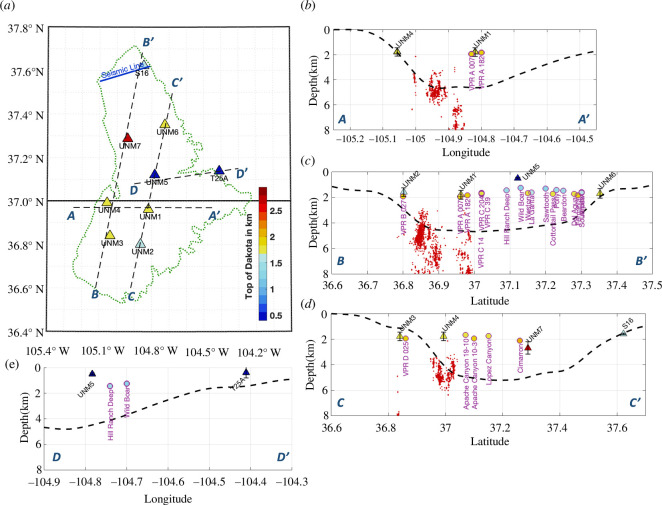
Comparison of H–κ stacking results with well data and large-scale sediment thickness map. (*a*) Map showing the broadband array, seismic line (from [62], see electronic supplementary material, figure S6) and near co-located wells with stratigraphic constraints (13; 14). (b–e) Cross-sections of profiles shown in (*a*): Top of the Precambrian basement (black dashed line) from Mooney & Kaban [45] and relocated earthquakes (red dots) within ~5 km of the profile lines. Triangles with error bars are H–κ stacking results from the broadband stations and circles are the top of the Dakota calculated from the elevation of the known top of Dakota formation from the wells with names labelled. Note that UNM5 is likely not reflecting Dakota (*e*) and no nearby seismicity was recorded.

Overall, we find that the top of the Dakota Formation is typically the most prominent source of P-to-S conversions, rather than the deeper boundary at the top of the crystalline basement. For example, at station UNM1, a well within 2 km (i.e. well VPR A 007) identified the top of the Dakota formation consistent with the 1.7-km layer thickness estimated from receiver function analysis (figure 5). At the northern edge of the Raton Basin, a prior two-dimensional controlled source survey and well data provide further opportunities for comparison with receiver function results from station S16 (figure 6). Reflection imaging of Applegate & Rose [62] identified the top of the Dakota as the most prominent interface and weaker underlying reflections that likely indicate the boundary between Paleozoic sedimentary strata and the underlying Precambrian basement. The reflection survey (electronic supplementary material, figure S6) and receiver functions from station S16 indicate a layer thickness of 1.6 km, which is consistent with the top of the Dakota from nearby well logs. Localized exceptions to the top of Dakota correlation include results from broadband station UNM5, just north of the NM-CO border in the centre of the basin, where the layer thickness estimated is only ~0.5 km, much shallower than the nearby well log constraints on the top of the Dakota formation at 1.46–1.62 km (figure 6). Given that UNM5 is closely surrounded by outcrops of mafic intrusions (see figure 1), we suggest that the shallower interface detected beneath UNM5 is related to Miocene and younger magmatic activity rather than sedimentary structures (figure 6*e*).

Due to the smaller inter-station distances, the node array receiver functions show evidence for finer structural variations, but it is not straightforward to relate all of them to topography on the top of the Dakota formation. Where broadband and node receiver functions are co-located, we observe strong agreement. For instance, UNM4 and a co-located node both yield depth to seismic interface estimates of 1.6 km. Similarly, broadband UNM1, a node, and a well all within 2 km of each other indicate the depth to the top of the Dakota is ~1.6–1.8 km (figure 5*a*). However, in some areas, there are large variations in the depth or layer thickness mapped with the node receiver functions. Near the western edge of the node array at latitudes 36.9° to 36.95°N, the receiver functions favour an interface only ~0.5 km deep (figure 5*a*). While a structural uplift may be expected due to the Tercio anticline, well data are unavailable to confirm whether ~1 km of relief on the top of the Dakota is realistic. Near the centre of the node array, at ~36.9°N, there is also an abrupt deepening of the velocity contrast detected by the node receiver functions, with five nodes near the La Veta syncline indicating depths of ~2.5 km (figure 5). Not all nodes along the strikes of the Tercio anticline or La Veta syncline show coherent changes in depth to the interface, so these results should be interpreted cautiously rather than as direct proxies for the depth to the top of the Dakota. The receiver functions could reflect different structural features at different locations, depending on the largest shallow velocity contrast that is locally present.

## 5. Interpretation and discussion

### (a) Variable behaviours between clusters and fault segments

Like in other injection settings, the near critically stressed, local-scale basement faults remained less known until a rise in seismicity rate in the past two decades [63]. Except for the currently quiescent 10-km-long Trinidad fault zone that hosted the M5.3 event, most of the recent seismicity in Raton Basin is suggested to occur on complex short fault segments [15, 48]. Our relocated seismicity from the nodal array further confirms that, similar to the segmented 10 km long Trinidad fault zone [17], the Vermejo Park fault zone comprises multiple <1-km-scale faults with variable internal geometry, as illustrated by C3 and C4 (see figure 2). Clear seismicity gaps exist between the clusters and suggest limited direct fluid connections. Combining our new observations and previous studies on seismicity locations, we suggest that the seismically active structures are more fractured than previously assumed across the entire Raton Basin. Such suggestions echo Riedel shear structures and *en echelon* faults around the recent injection-induced earthquakes in Alberta, Canada [8,64], as well as fracture-fault connections that have been observed around hydraulic fracturing [65,66]. The apparently high diffusivity (see figure 4) also echoes the sensitivity tested from a geomechanical modelling aspect by Stokes *et al*. [29], where they conclude that fault zone diffusivity is more influential on the seismicity distribution than variation in injection parameters between various wells within the basin. We believe C3 and C4 are the representative cases illustrating the dominating role for fluid pathways, with almost no injection wells around but consistently high seismicity rate. From this aspect, more detailed/complex fault architectures are encouraged for future modelling and risk predictions.

Distance between seismic cluster and injection wells, on the other hand, appears to be associated with the complexity (in both fault geometry and seismicity migration) of reactivated fault structure (figure 2). Comparing between the three active fault segments (i.e. C2–C4), the spatial–temporal evolutions of observed seismicity are also inconsistent: C3 and C4 show steady evolution along the fault strike and dip, whereas C2 reflects fault branching along a depth view (figure 4e). In addition, seismic energy within C2 is primarily released via deeper dip-slip events, opposite the expected normal regime within the basin. For such a less well-oriented vertical fault to slip, higher stress perturbation is expected. The expectation seems to be fulfilled at the location of C2: four injection wells within ~6 km (figure 2*a*). Vice versa, active injection is absent within such distances around C3 and C4, where only normal events are observed along more linearly defined hypocentre clusters. It is also worth noting that the southern Vermejo Park Fault zone has been active for more than a decade [59], mimicking a matured tectonic fault zone at large scale driven by earthquake–arthquake triggering [67].

### (b) Structural variations in the basin and potential links to basement fault reactivation

The identified seismic conversions from top of Dakota provide new views of depths to major velocity contrasts and earthquake hypocentre depths in Raton Basin. Previous investigation for other wastewater injection regions suggested that the distance between injection depths and basement controls the occurrences of induced seismicity [37,38] and might serve as a potentially seismological index factor [68,69]. Within the Raton Basin, injections are confined within the sedimentary layers above the Dakota Formation, shallower than 2 km and mostly between 1 and 1.5 km below the surface [15]. In contrast, almost all the induced events occurred within the crystalline basement (>3 km below surface), leaving a >1.5 km gap between injection and the seismically active depths across the basin. However, our receiver function results clearly indicate that the seismic contrast at the top of the Dakota formation is usually more prominent than that from the top of the basement. The prominent contrast could be associated with the change of rock types from the Dakota sandstone and Purgatoire formation to Sangre de Cristo formation below [70]. We speculate that high diffusivity structures (i.e. matured faults, dikes; electronic supplementary material, figure S6) exist within the deeper sediments and transmit elevated pore pressure to basement depths. In this case, the required downward migration distance is reduced to less than 2 km, making pore-pressure elevation more plausible within the crystalline, granite-dominated basement.

Finally, there is a ~2 km of topographic variation of the depth to a prominent velocity contrast near the La Veta syncline and fault segment C3, which are both within the node array (figure 5). Based on well data, it is improbable that there could be ~2 km of relief on the top of Dakota over distances of only a few km. However, the receiver function results from 4 to 5 nodes near the centre of the La Veta syncline coherently indicate an abrupt structural transition with the dominant seismic interface at locally greater depths near ~2.5 km (figure 5*a*). If the structural transition is not due to a fault offset of sedimentary strata, it could indicate another deep heterogeneity possibly of magmatic origin given the location between the ~27 and 22 Ma Spanish Peaks intrusions adjacent to the north [71] and primarily <10 Ma basaltic volcanism of the Raton–Clayton volcanic field adjacent to the south and east (e.g. [72]). The location with highest gradient in depth to the dominant velocity contrast roughly coincides with the east-dipping fault segment C3. Again, such abrupt changes could hint at heterogeneous structures that provide high fluid diffusivity pathways and promote reactivation of faults in C3, C4, as well as the surrounding scattered seismicity. We refrain from more detailed interpretation because the temporary nodal array only provided up to six events suitable for teleseismic receiver function analysis. We note that greater scrutiny of structural contrasts overlying the most active seismicity clusters of the Raton Basin may be warranted.

## 6. Conclusion

In this study, we analysed seismological monitoring records within the Raton Basin, employing two main approaches: first, we reexamined active earthquake clusters documented in the nodal-array catalogue; second, we refined the identification of basement reflectors. Our waveform cross-correlation-based earthquake relocations illuminate the active sub-kilometre-scale fault segments switching from east-dipping in the south- to west-dipping in the north, corroborated by focal mechanisms. The significant variation between the two adjacent faults along the southern end of Vermejo Park fault zone hints at the complexity of the sedimentary structures that may explain the heterogeneous distribution of induced seismicity. A greater diversity of active fault orientations is observed near the border between Colorado and New Mexico, where more injection wells are located and higher stress perturbation is expected. To better understand the communication between industrial fluids and subsurface geometries, we assessed subsurface structural interfaces using receiver functions and found that the top of the Dakota formation is the most prominent structure. Some abrupt variations in the depth to the dominant shallow velocity contrast are found near seismically active basement faults. These structural contrasts could indicate locally different lithology owing to the history of magmatism or offsets in the top of the Dakota formation. Such structural heterogeneities could provide pathways for fluid connectivity between the injection depth interval of ~1–1.5 km below the surface and seismicity that begins at ~3 km depth, where we estimate the top of the basement beneath the nodal array. Overall, our refined catalogue, source mechanisms and identified structural interfaces provide insights into how the basin structures influence the spatial variation of induced seismicity within the Raton Basin. As our work mainly collects and presents first-order seismological observations on both the sources and structures, future work is expected to fully use these well-constrained observations and may consider structure complexity in both geomechanical modelling and hazard evaluation.

## Data Availability

All data are available through EarthScope Data Services under network code 4E for nodal array [73], under network code YX for the seven UNM broadband stations [74] as well as the TA station T25A and S16. EarthScope Data Services facilities are funded through the Seismological Facility for the Advancement of Geoscience (SAGE) Award of the National Science Foundation under Cooperative Agreement EAR-1724509. Receiver Function analysis was performed using the open-source software package [54]. The initial and refined catalogue will be available at ISC upon publication [75]. All packages (PhaseNet, REAL, VELEST and hypoDD) are available through cited references [39,49,76,77].The wrapper (LOC-FLOW) package is maintained at [78]. Supplementary material is available online [79].
